# Histopathological correlates of haemorrhagic lesions on *ex vivo* magnetic resonance imaging in immunized Alzheimer’s disease cases

**DOI:** 10.1093/braincomms/fcac021

**Published:** 2022-02-03

**Authors:** Ashley A. Scherlek, Mariel G. Kozberg, James A. R. Nicoll, Valentina Perosa, Whitney M. Freeze, Louise van der Weerd, Brian J. Bacskai, Steven M. Greenberg, Matthew P. Frosch, Delphine Boche, Susanne J. van Veluw

**Affiliations:** 1 MassGeneral Institute for Neurodegenerative Disease, Massachusetts General Hospital/Harvard Medical School, Boston, MA, USA; 2 J. Philip Kistler Stroke Research Center, Massachusetts General Hospital/Harvard Medical School, Boston, MA, USA; 3 Clinical Neurosciences, Clinical and Experimental Sciences School, Faculty of Medicine, University of Southampton, Southampton General Hospital, Southampton, UK; 4 Department of Radiology, Leiden University Medical Center, Leiden, the Netherlands; 5 Department of Human Genetics, Leiden University Medical Center, Leiden, the Netherlands; 6 Neuropathology Service, C.S. Kubik Laboratory for Neuropathology, Massachusetts General Hospital/Harvard Medical School, Boston, MA, USA

**Keywords:** amyloid β, immunotherapy, cerebral amyloid angiopathy, siderosis

## Abstract

Haemorrhagic amyloid-related imaging abnormalities on MRI are frequently observed adverse events in the context of amyloid β immunotherapy trials in patients with Alzheimer’s disease. The underlying histopathology and pathophysiological mechanisms of haemorrhagic amyloid-related imaging abnormalities remain largely unknown, although coexisting cerebral amyloid angiopathy may play a key role. Here, we used *ex vivo* MRI in cases that underwent amyloid β immunotherapy during life to screen for haemorrhagic lesions and assess underlying tissue and vascular alterations. We hypothesized that these lesions would be associated with severe cerebral amyloid angiopathy. Ten cases were selected from the long-term follow-up study of patients who enrolled in the first clinical trial of active amyloid β immunization with AN1792 for Alzheimer’s disease. Eleven matched non-immunized Alzheimer’s disease cases from an independent brain brank were used as ‘controls’. Formalin-fixed occipital brain slices were imaged at 7 T MRI to screen for haemorrhagic lesions (i.e. microbleeds and cortical superficial siderosis). Samples with and without haemorrhagic lesions were cut and stained. Artificial intelligence-assisted quantification of amyloid β plaque area, cortical and leptomeningeal cerebral amyloid angiopathy area, the density of iron and calcium positive cells and reactive astrocytes and activated microglia was performed. On *ex vivo* MRI, cortical superficial siderosis was observed in 5/10 immunized Alzheimer’s disease cases compared with 1/11 control Alzheimer’s disease cases (*κ* = 0.5). On histopathology, these areas revealed iron and calcium positive deposits in the cortex. Within the immunized Alzheimer’s disease group, areas with siderosis on MRI revealed greater leptomeningeal cerebral amyloid angiopathy and concentric splitting of the vessel walls compared with areas without siderosis. Moreover, greater density of iron-positive cells in the cortex was associated with lower amyloid β plaque area and a trend towards increased post-vaccination antibody titres. This work highlights the use of *ex vivo* MRI to investigate the neuropathological correlates of haemorrhagic lesions observed in the context of amyloid β immunotherapy. These findings suggest a possible role for cerebral amyloid angiopathy in the formation of haemorrhagic amyloid-related imaging abnormalities, awaiting confirmation in future studies that include brain tissue of patients who received passive immunotherapy against amyloid β with available *in vivo* MRI during life.

## Teaser

Scherlek *et al.* report haemorrhagic lesions on *ex vivo* MRI in brain tissue samples of Alzheimer’s disease patients who received anti-amyloid immunotherapy during life. These lesions were associated with severe leptomeningeal cerebral amyloid angiopathy, suggesting a possible role for cerebral amyloid angiopathy in the formation of haemorrhagic amyloid-related imaging abnormalities.

## Graphical Abstract

**Figure:**
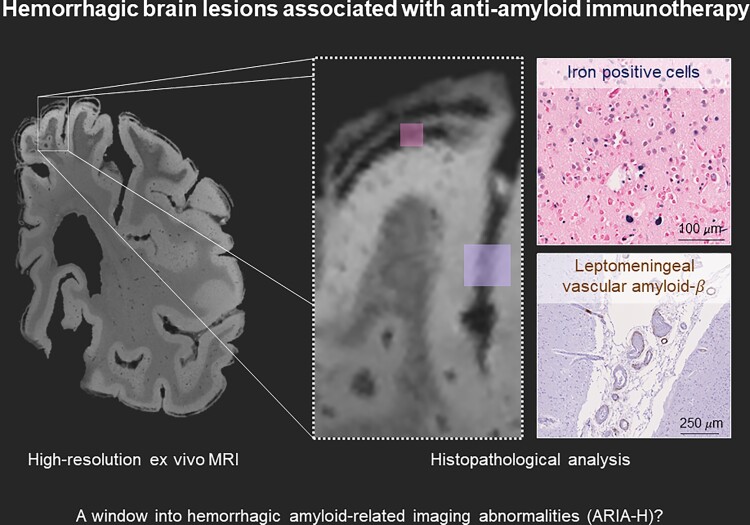

## Introduction

A prominent feature of Alzheimer’s disease is the accumulation of the amyloid β (Aβ) peptide in the brain in the form of plaques and cerebral amyloid angiopathy (CAA).^1^ Aggregation of Aβ has been suggested as an early event in the pathophysiology of Alzheimer’s disease, followed by the accumulation of hyperphosphorylated tau, and cognitive dysfunction. In this framework, intervention strategies have focused on anti-Aβ immunotherapy to halt a detrimental cascade of events. Long-term follow-up studies of the first active immunization trial (AN1792, Elan Pharmaceuticals Inc.) demonstrated that Aβ can be effectively removed from the brain.^2,3^ Subsequent passive immunotherapy trials similarly demonstrated Aβ clearance, culminating most recently in the US Food and Drug Administration’s approval of aducanumab for the treatment of Alzheimer’s disease. One of the major obstacles in the development of successful passive anti-Aβ immunotherapies has been the occurrence of adverse events including amyloid-related imaging abnormalities (ARIA) on brain MRI. ARIA can be observed in the form of signal hyperintensities on fluid-attenuated inversion recovery (FLAIR) thought to represent acute/subacute vasogenic oedema and/or sulcal effusion termed ARIA-E, and signal hypointensities on T_2_*-weighted gradient echo (GRE) MRI thought to represent haemosiderin deposits (e.g. microbleeds and cortical superficial siderosis) termed ARIA-H.^4–6^ When ARIA presents with symptoms, they can be managed with steroids and discontinuation of the anti-Aβ immunotherapy.^7^ The first active immunization trial against Aβ was halted because of the development of meningoencephalitis in 6% of participants, which shared similarities with ARIA although the term had not been introduced yet at the time.^8,9^ Subsequent passive immunotherapy trials have reported ARIA in up to 47% of participants receiving the highest antibody doses.^10,11^ Most recent data from the Phase 3 aducanumab trials indicated that ARIA-E was observed in 35% and ARIA-H in 19% (microbleeds) and 15% (cortical superficial siderosis) of patients treated with the highest dose (10 mg/kg).^12^

The neuropathological associations and pathophysiological mechanisms of ARIA are currently unknown, although the presence of CAA has been suggested as a major contributing factor.^6,13^ Approximately 50% of patients with Alzheimer’s disease have moderate-to-severe CAA on post-mortem examination of the brain.^14^ The pathophysiology of CAA is postulated to involve a feed-forward cycle of impaired perivascular Aβ drainage, subsequent accumulation of Aβ in the walls of cortical arterioles and leptomeningeal arteries, smooth muscle cell degeneration, impaired vascular compliance and further Aβ accumulation. Eventually, this results in vessel wall breakdown, extravasation of plasma proteins into the brain and haemorrhages.^1^ Brain haemorrhages in the form of lobar microbleeds, cortical superficial siderosis and symptomatic lobar intracerebral haemorrhage are the hallmark neuroimaging manifestations of CAA and form the basis of the diagnostic criteria for CAA during life.^15,16^ A subset of patients with CAA presents with spontaneous ARIA-like imaging abnormalities and clinical symptoms in the form of vasogenic oedema and haemorrhages, termed CAA-related inflammation. Analysis from Pittsburgh compound B (PiB)-PET imaging studies and CSF samples suggests that CAA-related inflammation may be the result of a spontaneous anti-Aβ immune response and immune-mediated Aβ removal from the brain.^7,17,18^ It remains unknown whether similar mechanisms play a role in the formation of ARIA in Aβ immunotherapy trials in Alzheimer’s disease patients.^19,20^ So far, neuropathological investigations of ARIA have been limited due to the scarcity of appropriate autopsy cases. To address this knowledge gap, we performed *ex vivo* MRI in formalin-fixed brain tissue samples of cases that underwent active immunization with AN1792 against Aβ to screen for possible ARIA-H and to perform a detailed neuropathological investigation of the underlying tissue and vascular abnormalities. Our investigation was limited to haemorrhagic lesions as the transient nature of ARIA-E precludes the assessment of these lesions *ex vivo*.

## Materials and methods

### Human brain tissue

Twelve cases with well-preserved formalin-fixed brain tissue from the long-term follow-up study of patients who enrolled in the first clinical trial of active Aβ immunization for Alzheimer’s disease (AN1792, Elan Pharmaceuticals Inc.) and provided consent for brain donation were included (iAD cases).^3^ Ethical approval was obtained through the Southampton and South West Hampshire Local Research Ethics Committees (REC reference 075/03/w). Brain tissue from 11 matched (for age, sex, fixation duration and Braak stage) non-immunized Alzheimer’s disease cases was included as ‘controls’ (cAD cases) from the South West Dementia Brain Bank (REC reference 18/SW/0029). One iAD case (Case #1) had documented ARIA during life^8^ and as such was considered a positive control case. Two out of 12 iAD cases received placebo, of which one (Case #14) proved not to have Alzheimer’s disease on neuropathological examination. As such, the placebo cases were excluded, and we considered the cAD cases to be the negative controls in this study.

Six additional non-Alzheimer’s disease control cases were included to control for long-term formalin fixation (Supplementary material).

From each case, one intact formalin-fixed coronal brain slice from the occipital lobe from a single cerebral hemisphere was selected for *ex vivo* MRI scanning. The occipital lobe was selected as CAA tends to be more severe in the posterior parts of the brain.

For comparison, formalin-fixed brain tissue of a case from the Massachusetts General Hospital stroke research centre with a clinical diagnosis of probable CAA and evidence of disseminated cortical superficial siderosis on *in vivo* MRI was added to the study.^21^ From this case, an intact formalin-fixed coronal brain slice from the occipital and frontal lobe from a single cerebral hemisphere was selected for *ex vivo* MRI scanning.

Detailed case characteristics are presented in Table 1 and Supplementary Table 1.

**Table 1:** Case characteristics

Case ID	Sex (M/F)	AN1792 dose (μg)	Injections (nr.)	Antibody response (mean titre)	Survival time from first immunization (months)	Dementia duration (years)	Age at death (years)	Time of death (year)	Cause of death (as per formal death certificate)	Neuropathology findings			Post-mortem interval (hours)	Braak stages	Evidence of plaque removal^a^	*APOE* ε4 allele (copy nr.)
										ABC scores	CAA	Other				
iAD																
102-1	F	50	5	119	20	6	74	2002	Pulmonary embolism, DVT, neurological disorder	A3B3C3	Numerous cortical microvascular lesions related to severe CAA	Meningoencephalitis	30	V/VI	++	1
102-6	M	50	8	1707	57	7	81	2005	Old age, Alzheimer’s disease	A3B3C3	Severe CAA		6	V/VI	++	1
102-7	M	50	8	4374	60	6	82	2005	Old age, Alzheimer’s disease, cerebral atrophy	A1B3C0			16	V/VI	+++	1
102-8	M	50	8	6470	64	10	63	2005	Ischaemic heart disease, coronary artery atherosclerosis	A1B3C1			6	V/VI	+++	1
102-11	M	50	8	142	94	12	88	2008	Bronchopneumonia and mucinous cystadenoma of pancreas (stented), dementia	A3B3C2	Severe CAA		8	V/VI	+	1
102-16	F	225	6	142	111	15	89	2009	Cerebrovascular accident, dementia	A3B3C0	Extensive capillary CAA	Small acute infarct left basal ganglia	Unk	V/VI	+++	1
102-17	F	50	Unk	0	141	13	86	2012	Bronchopneumonia, Alzheimer’s disease	A3B3C3		Small old right parietal infarcts, microvascular lesions entorhinal cortex	7	V/VI	+	2
102-20	M	225	3	430	166	17	82	2014	Alzheimer’s disease	A3B3C2	Extensive capillary CAA		15	V/VI	+++	Unk
102-21	F	50	8	3045	173	18	87	2014	Lower respiratory tract infection, dementia	A3B3C1	Extensive capillary CAA		96	V/VI	+++	1
102-22	M	225	Unk	1313	184	18	74	2015	Alzheimer’s disease	A3B3C3	Extensive capillary CAA		Unk	V/VI	+	2
102-14	M	Placebo	8	0	96	9	64	2008	Alzheimer’s disease	A3B1C0		TDP43+ inclusions, hippocampal sclerosis	10	I/II	−	2
102-15	F	Placebo	Unk	7	104	12	93	2009	Heart failure, ischaemic heart disease, diabetes mellitus, Alzheimer’s disease	A3B3C3	Moderate CAA	Severe arteriolosclerosis/rarefaction of cerebral white matter, several cortical microinfarcts	72	V/VI	−	1
cAD																
601	M	n/a	n/a	n/a	n/a	10	68	2000	Advanced Alzheimer’s disease	B3			61	VI	n/a	0
668	F	n/a	n/a	n/a	n/a	15	71	2001	Bronchopneumonia	B3			24	VI	n/a	2
674	M	n/a	n/a	n/a	n/a	3	81	2001	Unk	B3			24	VI	n/a	Unk
679	F	n/a	n/a	n/a	n/a	5	82	2001	Dementia	B3			110	IV	n/a	1
683	M	n/a	n/a	n/a	n/a	5	83	2002	Unk	B3C3			48	V	n/a	0
685	M	n/a	n/a	n/a	n/a	10	74	2001	Perforated benign oesophageal ulcer, bronchopneumonia	B3C2			48	V	n/a	0
703	F	n/a	n/a	n/a	n/a	9	83	2003	Unk	A3B3C2			48	V	n/a	1
705	F	n/a	n/a	n/a	n/a	10	82	2003	Unk	A3B3C3			Unk	V	n/a	1
745	F	n/a	n/a	n/a	n/a	15	84	2006	Sigmoid volvulus, bronchopneumonia	A3B3C3	Moderate CAA	Severe arteriolosclerosis	21	VI	n/a	0
763	F	n/a	n/a	n/a	n/a	9	80	2007	Bronchial pneumonia, fragility of old age, severe dementia	A3B3C3			51	V	n/a	2
765	M	n/a	n/a	n/a	n/a	3	80	2007	Bronchopneumonia	A3B3C2	Moderate CAA		24	IV	n/a	1
*CAA*																
CAA15	M	n/a	n/a	n/a	n/a	n/a	67	2017	Unk	A3B1C1	Severe CAA	Lobar haemorrhage right frontal lobe, remote microvascular lesions	24	I	n/a	Unk

^a^ Evidence of plaque removal was assessed according to previous methods detailed in Nicoll *et al.*^3^

### *Ex vivo* MRI

Three-to-four slices were simultaneously scanned for each MRI scan session. Slices were submerged in 10% formalin at room temperature in a glass container that fit in the head coil of the MRI scanner. Care was taken to avoid air bubbles by gently shaking the tissue. Scans were acquired overnight on a whole-body 7 T MRI scanner (Siemens Healthineers, MAGNETOM, Erlangen, Germany) with a custom-built 32-channel head coil. The scan protocol included a T_2_*-weighted GRE sequence (two averages each with three different flip angles were run with the following parameters: 10°, 20°, 30°, echo time 8.67 ms, repetition time 20 ms, voxel size 200 μm × 200 μm × 200 μm, scan duration ∼1 h per run) and a T_2_-weighted turbo spin echo (TSE) sequence (four averages were run with the following parameters: echo time 63 ms, repetition time 1000 ms, voxel size 300 μm × 300 μm × 300 μm, scan duration ∼1 h per run).

### *Ex vivo* MRI rating

Since ARIA-H has not been described on *ex vivo* MRI before, two raters (A.A.S., a trained rater with 2 years of experience and S.J.v.V., an expert rater with >8 years of experience) screened the acquired MR images first for the presence of haemorrhagic lesions. Hypointense signal abnormalities suggestive of cortical superficial siderosis^21,22^ were noted on the GRE scans in the form of curvilinear cortical hypointensities in both the positive control iAD case (Case #1) and several other iAD cases (but not the cAD cases). In addition, we noted occasional rarefaction of the juxtacortical white matter in the form of confluent hypointense abnormalities in iAD cases. No notable differences between iAD and cAD cases were observed in any of the other neuroimaging features on the TSE scans, such as ischaemic lesions, white matter hyperintensities or enlarged perivascular spaces. After this initial screening, the GRE MRI scans were de-identified and re-assessed by two raters (M.G.K. and S.J.v.V., both expert raters with 5–10 years of experience) blinded to treatment status and clinical information. The inter-rater agreement for the assessment of focal or disseminated cortical superficial siderosis was moderate (*κ* = 0.515), and for widespread (affecting >50% of the tissue) white matter rarefaction was perfect (*κ* = 1.000). Discrepancies were resolved in a consensus meeting, after which a final rating was established.

### Histopathology

In the iAD cases, areas with and without haemorrhagic lesions were sampled guided by *ex vivo* MRI and cut to fit a standard tissue cassette (measuring ∼30 × 25 × 4 mm). In the cAD and non-Alzheimer’s disease cases, at least one area was sampled at the level of the calcarine cortex, and additional areas if abnormalities were observed on MRI. Collectively, from the iAD cases, a total of 10 samples were taken from areas with haemorrhagic lesions suggestive of ARIA-H (in the five cases with siderosis on MRI) and a total of 12 samples were taken from random areas without haemorrhagic lesions (in five cases without and two cases with siderosis on MRI). From the cAD cases, a total of 16 samples were taken. From the non-Alzheimer’s disease cases, a total of seven samples were taken. Samples were processed and embedded in paraffin and cut in 6 μm thick serial sections on a microtome.

Adjacent sections were stained with haematoxylin and eosin (H&E), Luxol fast blue H&E (for myelin), Perls’ Prussian blue (for iron) and Von Kossa (for calcium) using standard histology protocols. Immunohistochemistry was performed against pan-Aβ (clone 6F/3D, Dako, 1:200, Cat# M0872), hyperphosphorylated tau (AT8, Invitrogen, 1:400, Cat# MN1020), glial fibrillary acidic protein (GFAP, Sigma, 1:1000, Cat# G9269), phagocytic microglia [cluster of differentiation 68 (CD68), Dako, 1:500, Cat# M0814] and fibrin(ogen) (Dako, 1:500, Cat# A0080). The standardized immunohistochemistry protocol included deparaffinization and rehydration of the tissue through xylene and graded ethanol series, blocking of endogenous peroxidase with 3% H_2_O_2_ (20 min), treatment with formic acid (only for Aβ, 5 min), antigen retrieval in heated citrate buffer (only for GFAP, fibrin(ogen) and AT8, 20 min), blocking with normal horse or goat serum (1 h), overnight incubation with the primary antibody at 4°C, incubation with a biotinylated mouse or rabbit secondary antibody (Vectastain ABC kit, Vector Laboratories, 1 h), followed by incubation with a mixture of avidin (A) and biotinylated HRP (B) (Vectastain ABC kit, Vector Laboratories, 30 min) and development with 3,3′-diaminobenzidine (Vector Laboratories). Sections were counter stained with haematoxylin (10 s), dehydrated through graded ethanol series and cover slipped with Fisher Chemical Permount mounting medium. In between steps, sections were washed with tris-buffered saline.

### Histopathological image analysis

All stained sections were scanned on a NanoZoomer whole slide scanner (Hamamatsu Photonics K.K., Japan) using a 20× objective, and the obtained digital images were assessed using the NDP.view2 viewing software (version 2.7.25).

Sections were de-identified and assessed by two independent raters (A.A.S. and S.J.v.V.) blinded to MRI findings, treatment status and clinical information, for the following: (i) number of microinfarcts on H&E and GFAP,^23^ (ii) number of microbleeds on H&E and Perls’,^23^ (iii) degree of concentric splitting of the wall of leptomeningeal blood vessels on H&E and Aβ-stained sections as absent, mild, moderate, severe (0–3)^24^ and (iv) tau severity on AT8-stained sections as absent, mild, moderate, severe (0–3). Final ratings were established in a consensus meeting.

In addition, we obtained artificial intelligence (AI)-assisted quantitative measurements using the online platform Aiforia®. The methodology is described in detail elsewhere.^25^ Briefly, digital images were uploaded to the platform, after which several convolutional neural networks were trained on manual annotations (drawn by AAS) of histopathological features of interest on a representative subset of sections. Once fully trained, the AI-models were applied to all sections (one section per stain per area). We obtained the following quantitative measurements: (i) percentage cortical tissue area occupied by Aβ plaques, (ii) percentage cortical tissue area occupied by CAA, (iii) percentage leptomeningeal area occupied by CAA, (iv) density of iron-positive deposits within the cortex, (v) density of calcium positive cells within the cortex, (vi) cell density of reactive (GFAP-positive) astrocytes within the cortex, (vii) cell density of activated/phagocytic (CD68-positive) microglia in the cortex and (viii) percentage cortical tissue area occupied by fibrin-positive vessels.

### Statistical analysis

To assess whether haemorrhagic lesions resulting from immunotherapy could be detected on *ex vivo* MRI, the presence or absence of *ex vivo* MRI abnormalities was compared between iAD and cAD cases with *χ*^2^ tests for proportions. Clinical and general neuropathological observations were compared between iAD cases with and without the presence of haemorrhagic lesions on *ex vivo* MRI with Mann–Whitney U-tests for non-parametric data. Histopathological and vascular alterations between samples from cAD and iAD cases and samples with and without haemorrhagic lesions from iAD cases were compared with Mann–Whitney U-tests and *χ*^2^ tests. Correlation analyses were performed with Spearman correlations. Cohen’s kappa was used to assess inter-rater reliability. All statistical analyses were performed with IBM SPSS version 22 and graphs were created with GraphPad Prism version 9.0.2. *P*-values of <0.05 were considered statistically significant. Statistics were performed on the number of samples rather than the number of cases.

### Data availability

The original data that support the findings of this study are available from the corresponding author upon reasonable request.

## Results

A total of 10 iAD cases (mean age at death 80.6 ± 8.1 years, four females) and 11 cAD cases (mean age at death 79.0 ± 5.4 years, six females) were included (Table 1). For the iAD cases, mean survival time from the first immunization was 107 ± 57 months and 8/10 died as a result of advanced Alzheimer’s disease dementia.

### *Ex vivo* 7T MRI findings

**Figure 1:**
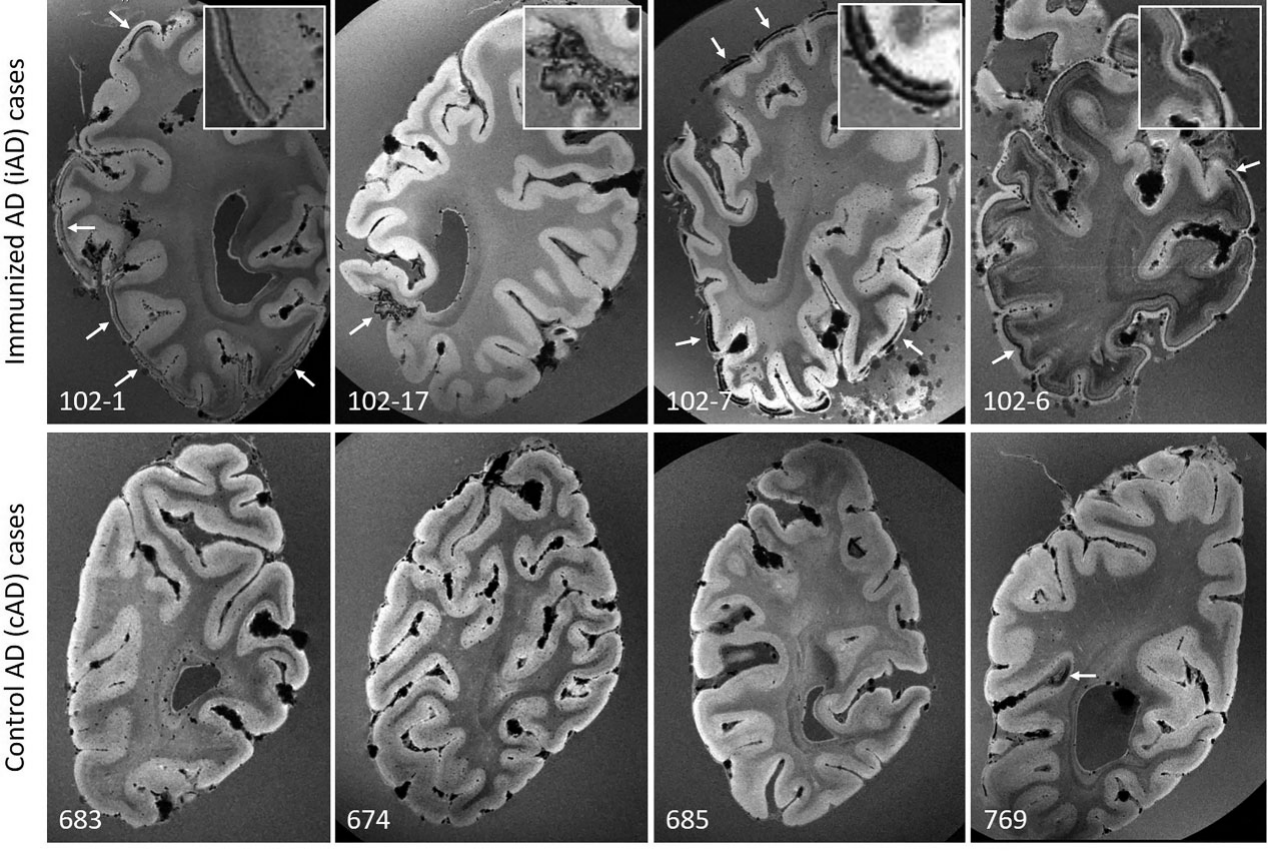
***Ex vivo* GRE 7T MRI reveals haemorrhagic lesions in iAD, but not cAD cases.** *Top* row shows examples of curvilinear cortical hypointensities (arrows) that were observed on *ex vivo* GRE 7T MRI scans of formalin-fixed coronal slices in five out of 10 iAD cases, representative of cortical superficial siderosis. Similar abnormalities were not observed in cAD cases (*bottom* row), except for a small area of cortical hypointensity in one case (arrow). Note the appearance of confluent hypointense abnormalities in the white matter in case 102-6, which may reflect tissue rarefaction related to formalin fixation, and was observed in 4 out of 10 iAD cases, but not in the cAD cases.

On the *ex vivo* GRE 7T MRI scan of iAD Case #1, who had documented ARIA during life 12 months before death,^8^ curvilinear hypointense abnormalities resembling cortical superficial siderosis were observed (Table 2 and Fig. 1). Similar abnormalities were observed in four other iAD cases, in the form of signal abnormalities resembling focal and disseminated cortical superficial siderosis. Such abnormalities were not observed in the cAD cases, except for one small area with subtle siderosis. Collectively, cortical superficial siderosis was observed in 5/10 iAD cases compared with 1/11 cAD cases (*χ*^2^ = 4.295, *P* = 0.038).

**Table 2:** *Ex vivo* 7T MRI findings

Case ID	Curvilinear cortical hypointensities on *ex vivo* GRE MRI	White matter rarefaction on *ex vivo* GRE MRI	Cortical microbleeds on *ex vivo* GRE MRI (nr.)	Cortical microinfarcts on *ex vivo* TSE MRI (nr.)	White matter hyperintensities on *ex vivo* TSE MRI	Perivascular spaces on *ex vivo* TSE MRI	Possible ARIA-H
iAD							
102-1	Yes, disseminated	No	0	0	Yes (including tissue loss)	Yes	Yes
102-6	Yes, disseminated	Yes	0	1	No	Yes, focally severe	Yes
102-7	Yes, disseminated	No	0	0	Yes	Yes, focally severe	Yes
102-8	No	Yes	0	0	Yes	Yes	No
102-11	No	Yes	0	0	No	Yes	No
102-16	Yes, disseminated	No	0	0	No	Yes	Yes
102-17	Yes, focal	No	0	0	No	No	Yes
102-20	No	No	0	0	Yes, severe	No	No
102-21	No	No	0	1	No	Yes	No
102-22	No	Yes	0	0	No	No	No
102-14	No	Yes	0	2	No	Yes, focally severe	No
102-15	No	No	0	0	No	Yes	No
cAD							
601	No	No	0	0	Yes	Yes, focally severe	No
668	No	No	0	0	Yes	No	No
674	No	No	0	2	No	Yes	No
679	No	No	0	0	Yes	Yes	No
683	No	No	0	0	Yes	Yes	No
685	No	No	0	1	No	No	No
703	No	No	0	0	Yes	Yes	No
705	No	No	0	0	Yes	Yes	No
745	No	No	1	6	Yes (including small infarct)	Yes	No
763	No	No	0	0	No	Yes	No
765	Yes, focal	No	0	0	Yes	Yes	Yes
CAA							
CAA15	Yes, disseminated	No	11^a^	8^a^	Yes	Yes	Yes

^a^ 7/11 microbleeds and 6/8 microinfarcts were found in the occipital slice.

One microbleed was found in one of the cAD cases (this case had moderate CAA on routine neuropathological examination, Table 1), and none were observed in the iAD cases.

On the *ex vivo* TSE 7T MRI scans, white matter hyperintensities and enlarged perivascular spaces were observed in 8/10 iAD cases and in 10/11 cAD cases (*χ*^2^ = 0.509, *P* = 0.476) (Supplementary Fig. 1). Cortical microinfarcts were observed in 2/10 iAD cases and 3/11 cAD cases (*χ*^2^ = 0.153, *P* = 0.696).

### Histopathological correlates of haemorrhagic lesions

**Figure 2:**
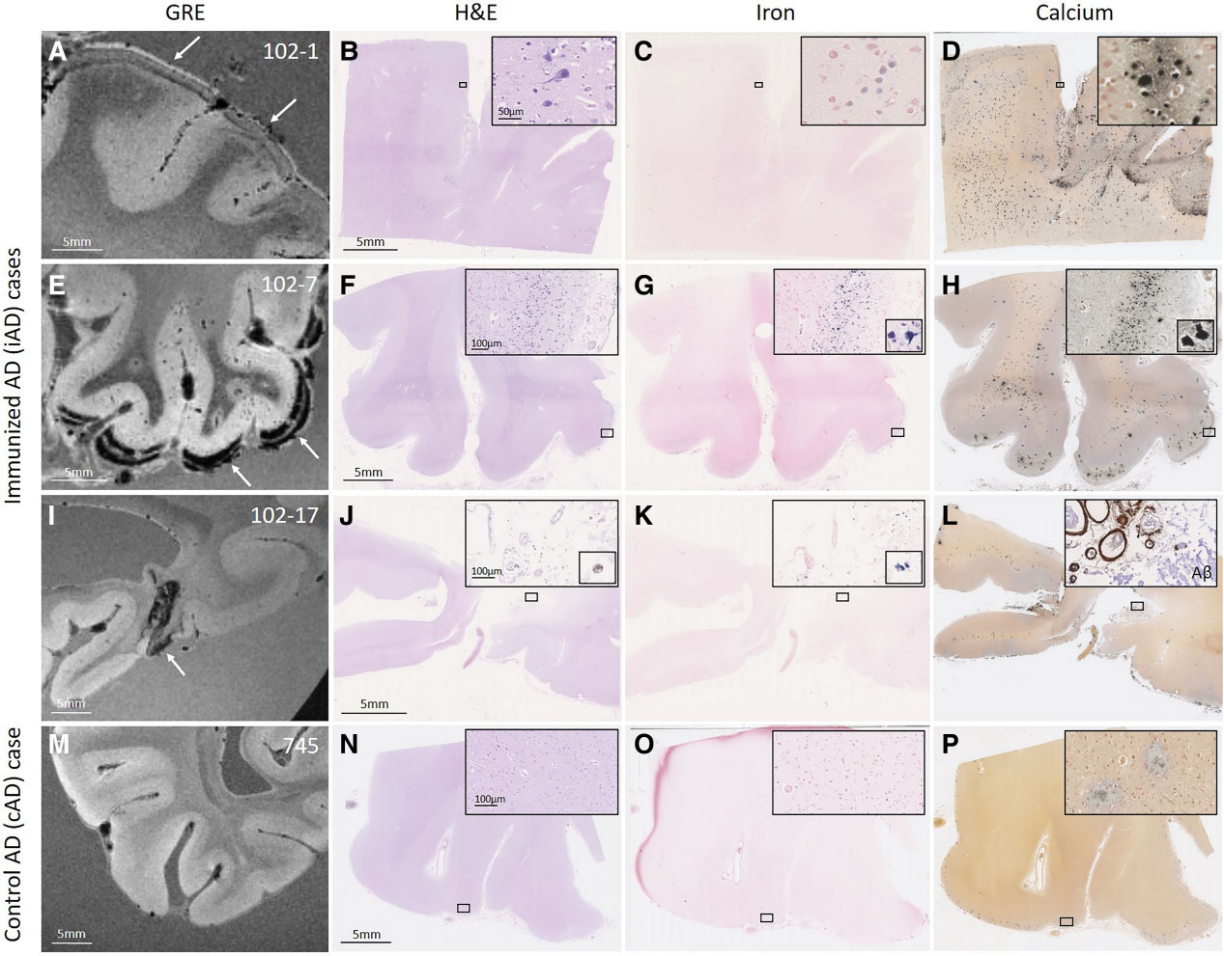
**Observed haemorrhagic lesions on *ex vivo* MRI correspond to iron and calcium deposits on histopathology.** An area with curvilinear cortical hypointensities (arrows) on *ex vivo* GRE 7T MRI in positive control iAD Case #1 (**A**) revealed on histopathology tissue abnormalities in the cortex on H&E (**B**), which were confirmed as intracellular iron-positive depositions on an adjacent Perls’ Prussian blue-stained section (**C**) and intracellular calcium depositions on an adjacent Von Kossa-stained section (**D**). In another iAD case with a more severe degree of cortical hypointensities (**E**, arrows), the corresponding histopathological sections revealed more extensive tissue abnormalities (**F**), in the form of intracellular iron depositions (**G**) and intracellular calcium depositions (**H**). In another iAD case with focal cortical hypointensity (**I**, arrow), the corresponding histopathological sections revealed haemosiderin deposits at the level of the leptomeningeal vessels (**J**), which stained positive for iron (**K**) and calcium (**L**). The inset in (**L**) shows extensive leptomeningeal CAA as well as concentric vessel wall splitting as observed on an adjacent section that underwent immunohistochemistry against Aβ. The *bottom* row shows a representative example of one of the cAD cases, without abnormalities on *ex vivo* GRE 7T MRI (**M**), H&E (**N**), Perls (**O**) or Von Kossa-stained sections (**P**). Note the presence of extracellular calcium depositions in (**D, H, L, P**), which may be the result of prolonged formalin fixation of banked tissue.

Qualitative assessment revealed that the curvilinear hypointense signal abnormalities corresponded to iron-positive cellular and vascular deposits on Perls’ stained sections (Fig. 2). The intracellular deposits also stained positive for calcium. Notably, brown haemosiderin deposits on H&E suggestive of more recent (i.e. shortly pre-mortem) haemorrhage were only observed in the iAD case with focal cortical superficial siderosis on *ex vivo* MRI (iAD Case #17). Similar histopathological abnormalities were not present in the cAD cases, except for occasional extracellular calcium deposits in the cortex, which may be the result of long formalin fixation (see Supplementary material and Supplementary Fig. 3).

To help assess whether these findings are consistent with cortical superficial siderosis, we also analysed a brain with pathologically confirmed CAA and evidence of disseminated cortical superficial siderosis during life (Supplementary Fig. 2). *Ex vivo* MRI findings revealed widespread curvilinear hypointense abnormalities, extending more deeply into the sulci compared with iAD cases. Histopathological observations in areas with cortical superficial siderosis on *ex vivo* GRE 7T MRI in this case confirmed previously reported findings of haemosiderin and iron deposits in the cortex in the context of severe CAA and concentric splitting of nearby leptomeningeal vessel walls.^21^ Adjacent sections stained positive for intracellular calcium deposits in the cortex, whereas no extracellular calcifications were observed in either the cortex or white matter.

### Clinical and general neuropathological correlations of haemorrhagic lesions

Within the iAD group, the presence of cortical superficial siderosis on *ex vivo* MRI was not related to AN1792 dose, number of injections, antibody response, age at death, *APOE* ε4 status or post-mortem interval (Mann–Whitney U-tests, all *P*-values > 0.3) (Table 1).

### Quantification of histopathological alterations in iAD vs. cAD cases

**Figure 3:**
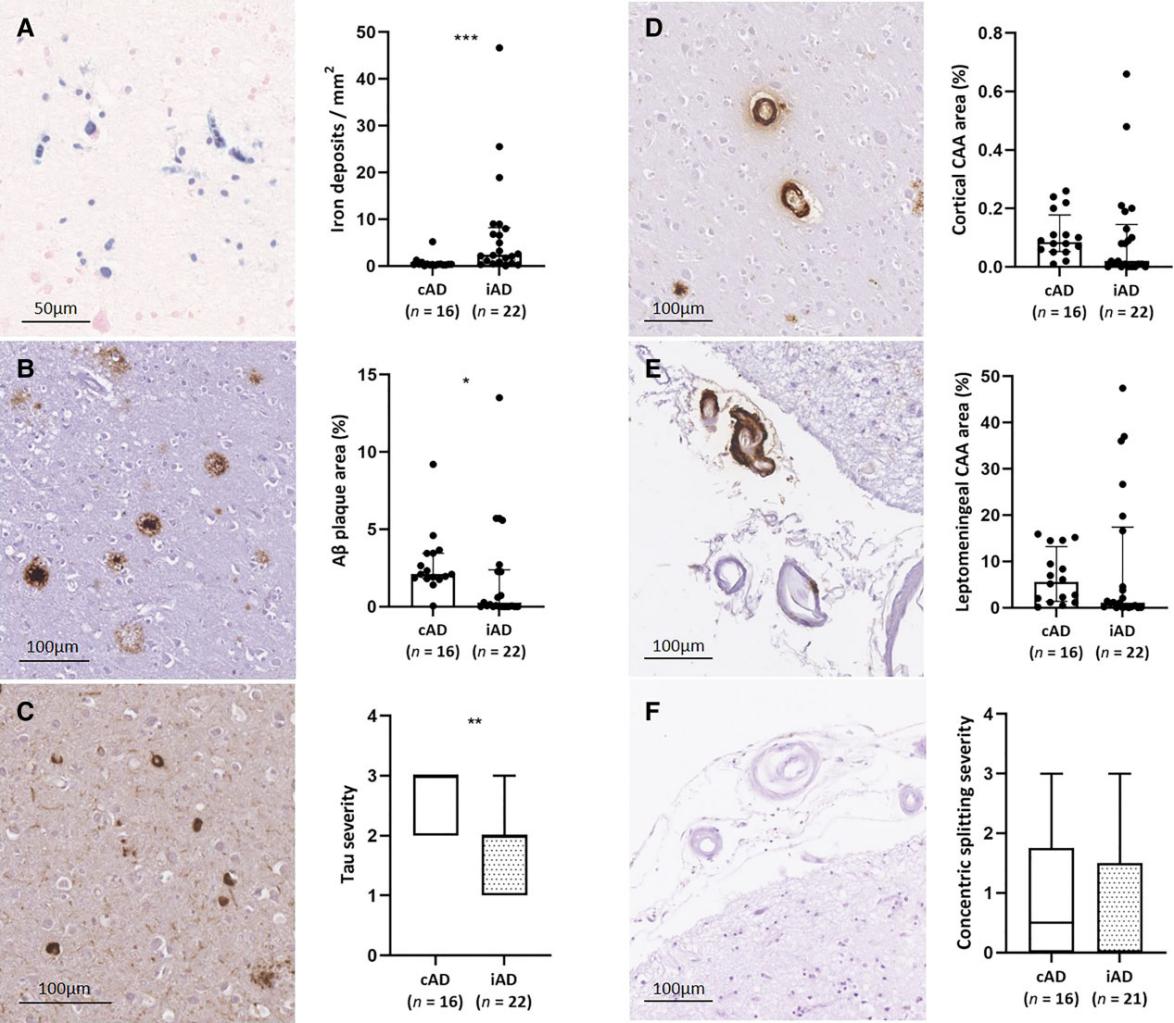
**Histopathological comparison between samples retrieved from iAD and cAD cases.** A greater density of iron deposits was observed in the cortex in iAD cases (*n* = 22 samples in 10 cases) compared with cAD cases (*n* = 16 samples in 11 cases) (**A**, *U* = 55, *P* < 0.001). Aβ plaque area was lower in the cortex in iAD compared with cAD cases (**B**, *U* = 97, *P* = 0.019). Tau severity was lower in the cortex in iAD compared with cAD cases (**C**, *U* = 72, *P* = 0.002). Percentage cortical CAA was comparable between iAD and cAD cases (**D**, *U* = 120, *P* = 0.101), as was leptomeningeal CAA area (**E**, *U* = 130, *P* = 0.181), and severity of concentric vessel wall splitting (**F**, *U* = 141, *P* = 0.421). Box plots represent median with interquartile range. Statistics were performed with Mann–Whitney U-tests. *P < 0.05, **P < 0.01, ***P < 0.001.

Using AI-assisted quantifications of Aβ, iron and calcium on digitized histopathological images,^25^ we found a greater density of intracellular iron and calcium deposits in the cortex in iAD cases (*n* = 22 samples) compared with cAD cases (*n* = 16 samples) (iron: *U* = 55, *P* < 0.001; calcium: *U* = 47, *P* < 0.001). Aβ plaque area (*U* = 97, *P* = 0.019) and tau severity (*U* = 72, *P* = 0.002, which was assessed semi-quantitatively) were both significantly lower in iAD compared with cAD samples, which is in line with previous reports in this cohort^3^ (Fig. 3).

In terms of vascular alterations, percentage cortical CAA (*U* = 120, *P* = 0.101) and leptomeningeal CAA area (*U* = 130, *P* = 0.181) did not differ between iAD (*n* = 22 samples) and cAD cases (*n* = 16 samples). Moreover, severity of concentric vessel wall splitting (which was assessed semi-quantitatively) was comparable as well (*U* = 141, *P* = 0.421) (Fig. 3).

### Quantification of histopathological alterations in areas with and without haemorrhagic lesions

**Figure 4:**
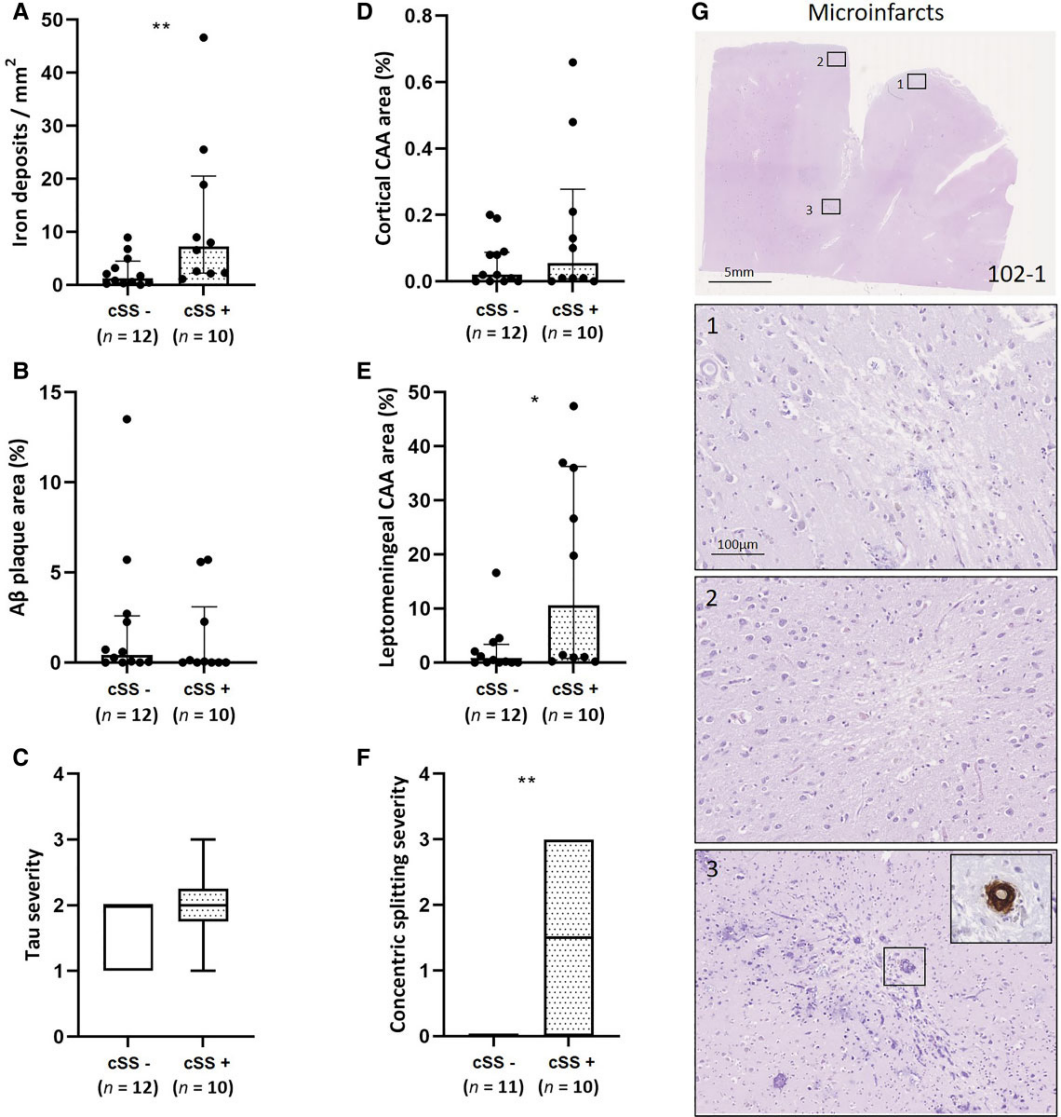
**Histopathological comparison between areas with and without haemorrhagic lesions in iAD cases.** A greater density of iron deposits was observed in cortical areas of iAD cases with haemorrhagic lesions (i.e. cortical superficial siderosis) on *ex vivo* MRI (*n* = 10 samples) compared with cortical areas of iAD cases without haemorrhagic lesions on *ex vivo* MRI (*n* = 12 samples) (**A**, *U* = 21, *P* = 0.009). Aβ plaque area (**B**, *U* = 45, *P* = 0.346) and tau severity (**C**, *U* = 44, *P* = 0.314) did not differ between areas with and without haemorrhagic lesions. Percentage cortical CAA was comparable between areas with and without haemorrhagic lesions (**D**, *U* = 47, *P* = 0.418). We found greater leptomeningeal CAA area (**E**, *U* = 26, *P* = 0.025) as well as degree of concentric vessel wall splitting (**F**, *U* = 22, *P* = 0.020) in areas of iAD cases compared with areas of iAD cases without haemorrhagic lesions. Note that in one sample, no leptomeningeal vessels were present to assess (**F**). The vast majority of microinfarcts were observed in positive control iAD case #1 (**G**). The insets show three representative examples of cortical microinfarcts on H&E. The inset in (3) shows a CAA-positive cortical vessel associated with the microinfarct. Box plots represent median and interquartile ranges. Statistics were performed with Mann–Whitney U-tests. *P < 0.05, **P < 0.01.

We next compared histopathological quantifications in areas with and without haemorrhagic lesions (i.e. cortical superficial siderosis) on *ex vivo* MRI in the iAD group. We found a greater density of iron deposits in areas with (*n* = 10 samples) compared with areas without haemorrhagic lesions (*n* = 12 samples) (*U* = 21, *P* = 0.009), but not intracellular calcium deposits (*U* = 37, *P* = 0.140). Aβ plaque area (*U* = 45, *P* = 0.346) and tau severity (*U* = 44, *P* = 0.314) did not differ between areas with and without haemorrhagic lesions (Fig. 4).

Regarding vascular alterations, percentage cortical CAA area was comparable between areas with (*n* = 10 samples) and without haemorrhagic lesions (*n* = 12 samples) (*U* = 47, *P* = 0.418). However, we found greater leptomeningeal CAA area (*U* = 26, *P* = 0.025) as well as degree of concentric vessel wall splitting (*U* = 22, *P* = 0.020) in areas with compared with areas without haemorrhagic lesions (Fig. 4). No relationship was found between haemorrhagic lesions and fibrin-positive vessels in the cortex (*U* = 45, *P* = 0.346).

### Microinfarcts and microhaemorrhages

A total of 21 microinfarcts were found in 6/22 samples of the iAD cases, compared with a total of 22 microinfarcts in 4/16 samples of the cAD cases (*χ*^2^ = 0.025, *P* = 0.875). Although there was no overall difference between the presence of microinfarcts in areas with and without haemorrhagic lesions (*χ*^2^ = 0.489, *P* = 0.484), 17/21 microinfarcts were observed in iAD Case #1 in areas with haemorrhagic lesions on *ex vivo* MRI (Fig. 4). While some degree of perivascular haemosiderin was occasionally present, frank microbleeds as observed in cases with severe CAA^23^ were absent on histopathology.

### Iron deposition is associated with lower Aβ plaque area

**Figure 5:**
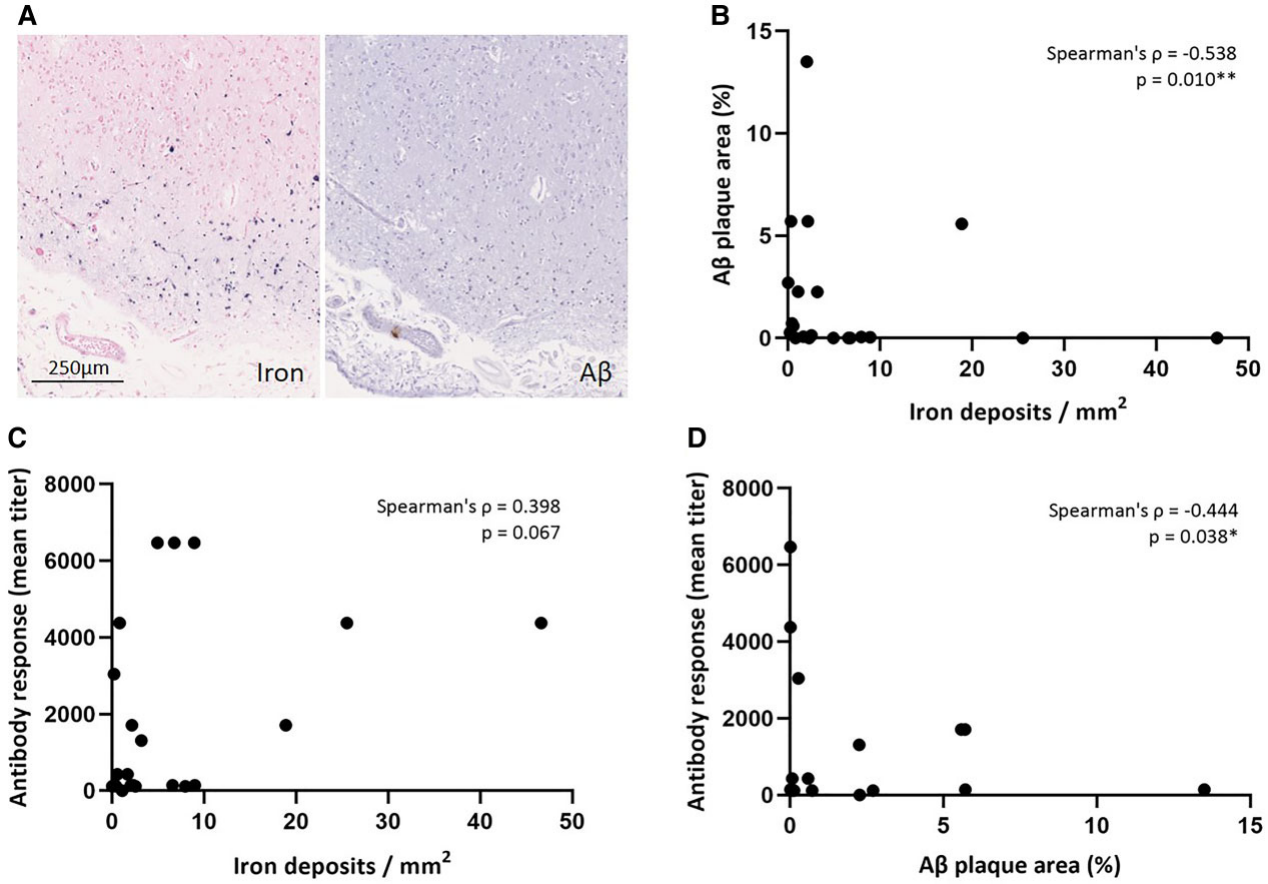
**Cortical iron deposition is associated with lower Aβ plaque area in iAD cases.** Iron-positive depositions were often found in areas with evidence of Aβ plaque removal as shown here in iAD Case #7 (**A**). Within the iAD group (*n* = 22 samples), the density of intracellular iron deposits in the cortex was negatively correlated with percentage Aβ plaque area in the cortex (**B**, Spearman’s *ρ* = −0.538, *P* = 0.010). The density of intracellular iron deposits in the cortex tended to be positively correlated with post-vaccination anti-AN1792 antibody titres (**C**, Spearman’s *ρ* = 0.398, *P* = 0.067). Percentage Aβ plaque area in the cortex was negatively associated with post-vaccination anti-AN1792 antibody titres (**D**, Spearman’s *ρ* = −0.444, *P* = 0.038). *P < 0.05, **P = 0.01.

Correlation analysis within the iAD group revealed that greater density of iron deposits in the cortex was associated with lower Aβ plaque area (*n* = 22 samples, Spearman’s *ρ* = −0.538, *P* = 0.010). Moreover, higher post-vaccination anti-AN1792 antibody titres were associated with lower Aβ plaque area (Spearman’s *ρ* = −0.444, *P* = 0.038) and tended to be associated with greater density of iron deposits in the cortex (Spearman’s *ρ* = 0.398, *P* = 0.067) (Fig. 5). Collectively, these findings suggest that haemorrhagic lesions in the context of active immunization against Aβ may be related to successful Aβ removal from the parenchyma.

### Increased survival time is associated with increased reactive cells

Notably, correlation analysis within the iAD group revealed that longer survival times were associated with greater densities of reactive astrocytes (Spearman’s *ρ* = 0.672, *P* = 0.001) and phagocytic microglia in the cortex (Spearman’s *ρ* = 0.788, *P* < 0.001) (Supplementary Fig. 4).

## Discussion

This study resulted in several main findings. We observed haemorrhagic lesions on *ex vivo* MRI in cases who received active immunization against Aβ during life. The *ex vivo* MRI and histopathological characteristics were consistent with cortical superficial siderosis, a known manifestation of severe CAA.^21^ Neuropathological examination confirmed locally severe vascular pathology of the leptomeningeal blood vessels, including greater vascular Aβ deposition and vessel wall splitting. The severity of cortical superficial siderosis was associated with lower Aβ plaque area and higher post-vaccination antibody titre levels. Finally, we observed greater presence of reactive glial cells in iAD cases with longer survival times.

Without *in vivo* MRI, we were not able to verify whether the haemorrhagic lesions on *ex vivo* MRI indeed corresponded to ARIA-H detected during life. Of note, similar hypointense signals on *ex vivo* MRI have been observed in post-mortem cases with early-onset Alzheimer’s disease^26^ and were observed in our current study in one non-Alzheimer’s disease control case. Moreover, another *ex vivo* MRI study in CAA patients identified similar hypointense stripes in the cortex, in these cases reflecting severe ferrugination in the form of iron and calcium depositions in cortical blood vessels.^27^ Taken together, the *ex vivo* MRI findings in iAD cases are not specific, although the underlying neuropathology in these areas (including increased leptomeningeal CAA severity and vascular remodelling) was suggestive of ARIA-H.

The term ARIA refers to a spectrum of image abnormalities that can be observed throughout the natural course of Alzheimer’s disease and CAA and in the setting of amyloid-modifying therapeutic approaches.^6^ Two subtypes have been defined based on *in vivo* MR imaging characteristics; ARIA-E (cerebral oedema and sulcal effusions on FLAIR MRI) and ARIA-H (cortical and superficial haemorrhages on T_2_*-weighted MRI).^6^ Both subtypes were initially observed in active immunization trials and were defined as subacute meningoencephalitis,^9^ and have now also been reported in multiple passive immunotherapy trials with rates of up to ∼40% in the Phase 1b and Phase 3 aducanumab^11,12^ and Phase 2 donanemab trials.^28^ ARIA is usually asymptomatic with symptoms reported in 1–26% of patients with ARIA-E.^1,12^ Notably, the increased rate of ARIA-H in patients treated with passive immunotherapy as compared with placebo is more modest than differences in ARIA-E^29^, an effect largely driven by higher rates of pre-existing microbleeds in Alzheimer’s disease cases. In our study, we observed abnormalities suggestive of ARIA-H on *ex vivo* MRI in 5/10 iAD cases as compared with 1/11 cAD cases. Important risk factors for the development of symptomatic ARIA include immunotherapy dose, *APOE* ε4 allele status, and baseline number of cerebral microbleeds, with higher doses and/or faster up-titration of antibody and two copies of the *APOE* ε4 allele associated with increased incidence of ARIA.^1^ We did not find a relationship between haemorrhagic lesions and *APOE* ε4 status in the current study, which may be because all iAD cases had at least one allele as well as the modest sample size.

CAA has been proposed as a potential driver of ARIA formation, supported by shared risk factors between CAA and ARIA including *APOE* ε4 allele status and number of microbleeds as well as similarities between ARIA- and CAA-related inflammation. Additionally, prior neuropathology findings have suggested increased CAA in iAD cases as well as increased concentric vessel wall splitting (i.e. lumen within a lumen appearance) detected in the frontal, temporal and parietal lobes.^30,31^ We did not observe similar differences in our study between iAD and cAD cases. As we included brain slices from the occipital lobe, in which more severe CAA is typically observed, we suspect that any relative increase in CAA in the iAD cases may not have been detectable because of the higher baseline CAA severity in cAD cases in these occipital areas.

Our ‘positive control’ (iAD Case #1) had symptomatic ARIA-E during life after receiving active Aβ immunization, confirmed by *in vivo* T_2_-weighted imaging.^8^*In vivo* T_2_*-weighted imaging was not available for this patient, which precluded us from verifying the presence of cortical superficial siderosis or other types of ARIA-H post-vaccination. An extensive neuropathological study of this case was previously published, demonstrating regions with likely Aβ plaque removal with relative persistence of CAA as well as T-lymphocyte meningoencephalitis.^8^ Of note, this was the only case in this cohort observed to have fulminant meningoencephalitis at autopsy.^3^ This case had the shortest interval between first immunization and death, possibly reflecting the severity of the adverse event. However, whereas other cases who received active immunization did not have any evidence of active meningoencephalitis at autopsy, neuropathological examination did provide evidence for Aβ plaque removal and increased CAA.^2,3^

Our findings extend this literature by adding the first direct histopathological investigation of haemorrhagic lesions in the context of Aβ immunotherapy. Without available *in vivo* MRI scans to verify whether these lesions indeed met the criteria for ARIA-H during life, our findings may not be generalizable. The MRI characteristics were suggestive of cortical superficial siderosis as frequently observed in cases with severe CAA and one of the subtypes of ARIA in Aβ immunotherapy trials. Cortical superficial siderosis in CAA patients represents the chronic phase of convexity subarachnoid haemorrhage and increases the risk of future haemorrhagic stroke.^32^ Our histopathological findings are in line with previous observations in CAA autopsy cases with documented cortical superficial siderosis during life and are indicative of bleeding from the leptomeningeal blood vessels in the context of severe CAA.^21^ Notably, we previously reported increased leptomeningeal (but not cortical) CAA severity and concentric splitting of the walls (i.e. lumen within a lumen appearance) of leptomeningeal vessels in areas with siderosis on histopathology,^21^ resembling our observations in iAD cases. Moreover, siderosis was associated with increased numbers of cortical microinfarcts, thought to be the result of secondary injury after leptomeningeal bleeding, which is in line with our findings in iAD Case #1.

Although the cross-sectional nature of this investigation does not allow us to infer causes and consequences, potential mechanisms that could explain our findings in iAD cases are proposed as follows: perivascular drainage of Aβ from the cortex after immunization may have resulted in increased deposition of Aβ in leptomeningeal arteries,^30^ resulting in vessel wall remodelling and fragmentation (in the form of vessel wall splitting)^31^ and subsequent leakage of blood products into the underlying cortex. An alternative interpretation of our findings is that the intraneuronal iron depositions observed in iAD cases are the result of released iron from local Aβ plaques after successful Aβ plaque removal. Previous studies have indeed demonstrated that Aβ plaques contain iron.^33–36^ In this scenario, our observations would not be related to bleeding and would likely be irrelevant to ARIA-H as observed during treatment trials. Although we cannot rule out this explanation, it does not account for the observed association with severe leptomeningeal CAA and concentric vessel wall splitting. Future studies, including longitudinal *in vivo* imaging studies in mouse models are needed to tease out the individual steps and exact sequence of events in ARIA formation.^37^

We found that longer survival times were associated with increased number of reactive glial cells, while previous studies reported an overall downregulation of neuroinflammatory protein expression following Aβ removal.^38,39^ This observation is detected with the addition of cases with longer post-immunization survival times and thus could reflect that the response to ongoing neurodegenerative processes, such as tau pathology, is still progressing.^3,13^ Future studies are needed to determine the nature of these neuroinflammatory responses.

The observation of extracellular calcifications in the tissue with accompanying hypointensities on *ex vivo* GRE MRI in the iAD cases may reflect artefacts due to prolonged formalin fixation. A previous study reported similar-appearing granular neuropil changes on H&E in tissue that was fixed for more than 6 years.^40^ These alterations were also present in non-Alzheimer’s disease control cases, further suggesting these are irrelevant to ARIA-H. Moreover, similar abnormalities were not found in tissue from the same iAD cases embedded in paraffin after weeks of fixation. Importantly, Von Kossa staining of these samples embedded within weeks of fixation did demonstrate intracellular calcium deposits in the cortex in one of the most severely affected iAD cases (Case #7). The adjacent iron-stained section was also positive, but the signal was notably less strong than the iron-stained sections in the tissue of the same case after years of fixation. One explanation for this apparent mismatch may be that formalin fixation augments endogenous intracellular iron and calcium depositions. However, reports in the literature that have examined the effect of formalin fixation on the concentration of minerals, including iron, either suggest no effect,^41^ or significantly lower iron levels in formalin fixed compared with fresh tissue.^42^ Alternatively, the absence of *in vivo* or *ex vivo* MRI to guide sampling in previous studies may explain why these tissue alterations had not been observed before in this dataset and further underlines the importance of *ex vivo* MRI to bridge the gap between observations made during life and neuropathology. It is unclear why similar amounts of extracellular calcifications were not found in the cAD cases that were matched for years of fixation and stored under similar conditions as the iAD cases. We therefore cannot completely rule out the possibility that some of the white matter rarefaction in iAD cases reflects old oedema in the setting of ARIA-E.

Besides the abovementioned caveats, this study has several additional limitations. First, because of the small number of cases that were available, we considered individual tissue blocks as independent samples. This can be considered a limitation given the unequal distribution of samples across cases. Second, only occipital brain slices were included in this study and it remains unclear whether similar alterations occur in other parts of the brain in which CAA is generally less severe. Third, because of the lack of established rating criteria for ARIA-H on *ex vivo* MRI, an initial unblinded screen was performed prior to blinded assessment by the same rater. Given the small sample size, this may have resulted in unintentional bias. Furthermore, rating *ex vivo* MRI scans remains challenging due to distortions (e.g. air trapped between stacked slices), which was reflected by a moderate inter-rater agreement. Of note, all neuropathological examinations were performed in areas where both raters agreed on the presence or absence of siderosis on *ex vivo* MRI locally. Fourth, since we did not have access to autopsy samples of patients who underwent passive immunotherapy during life, our findings may not be generalizable to anti-Aβ antibody treatments and ongoing studies that rely on passive immunotherapy.

## Conclusions

This study demonstrates the added value of using *ex vivo* MRI to screen for ARIA-H. With the abovementioned significant limitations in mind, our findings suggest that Aβ immunotherapy may result in chronic cortical superficial siderosis in the context of severe leptomeningeal CAA and vascular remodelling. Whether secondary tissue injury caused by cortical superficial siderosis such as cortical microinfarcts and neuronal mineralization may contribute to further cognitive decline in Alzheimer’s disease patients with ARIA-H needs to be assessed in future studies. Collectively, the screening, clinical management and ideally prevention of ARIA-H during Aβ immunotherapy trials are potentially relevant to limiting clinical deterioration, especially in Alzheimer’s disease patients with co-occurring CAA or *APOE* ε4 carriers who are at increased risk.^43^

## Supplementary Material

fcac021_Supplementary_Data

## Abbreviations

Aβ =: amyloid β
AI =: artificial intelligence
APOE =: apolipoprotein
ARIA =: amyloid-related imaging abnormalities
CAA =: cerebral amyloid angiopathy
CD68 =: cluster of differentiation 68
FLAIR =: fluid-attenuated inversion recovery
GFAP =: glial fibrillary acidic protein
GRE =: gradient echo
H&E =: haematoxylin and eosin
PiB =: Pittsburgh compound B
TSE =: turbo spin echo
