# Supplementary material for: Histopathological correlates of haemorrhagic lesions on ex vivo magnetic resonance imaging in immunized Alzheimer’s disease cases
Source: Brain Commun. 2022 Feb 3;4(1):fcac021. doi: 10.1093/braincomms/fcac021 (PMC8870423; doi:10.1093/braincomms/fcac021)
Supplement: fcac021_Supplementary_Data [file fcac021_supplementary_data.pdf]

SUPPLEMENTARY MATERIAL

Title: Histopathological correlates of hemorrhagic lesions on ex vivo MRI in immunized Alzheimer's disease cases

Running Title: Histopathology of ARIA-H

Ashley A. Scherlek[1], Mariel G. Kozberg[1,2], James A.R. Nicoll[3], Valentina Perosa[2], Whitney M. Freeze[4], Louise van der Weerd[4,5], Brian J. Bacskai[1], Steven M. Greenberg[2], Matthew P. Frosch[6], Delphine Boche[3], Susanne J. van Veluw[1,2,4*]

[1] *MassGeneral Institute for Neurodegenerative Disease, Massachusetts General Hospital / Harvard Medical School, Boston, MA, USA*

[2] *J. Philip Kistler Stroke Research Center, Massachusetts General Hospital / Harvard Medical School, Boston, MA, USA*

[3] *Clinical Neurosciences, Clinical and Experimental Sciences School, Faculty of Medicine, University of Southampton, Southampton General Hospital, Southampton, UK*

[4] *Department of Radiology, Leiden University Medical Center, Leiden, the Netherlands*

[5] *Department of Human Genetics, Leiden University Medical Center, Leiden, the Netherlands*

[6] *Neuropathology Service, C.S. Kubik Laboratory for Neuropathology, Massachusetts General Hospital / Harvard Medical School, Boston, MA, USA*

* Corresponding author: Susanne J. van Veluw, MassGeneral Institute for Neurodegenerative Disease, Massachusetts General Hospital, 114 16th street, Charlestown, 02129 MA, USA, svanveluw@mgh.harvard.edu

Supplemental Materials and Methods

Occipital brain slices from an additional six control autopsy cases without known neurological disease at time of death that were stored for >20 years in formalin under the same conditions as in SWDBB were sourced from BRAIN UK and included at a later stage of the study (REC reference 19/SC/0217). These cases did not have any evidence of AD pathology at neuropathological examination and are referred to as non-AD cases (Supplemental Table 1). These non-AD cases were included to assess whether any information obtained from the MRI scanning or from the neuropathological examination on the iAD cases was the consequence of ARIA or long-term fixation in formalin.

Supplemental Results

In addition, the GRE scans revealed widespread white matter rarefaction in 4/10 iAD cases compared to 0/11 cAD cases ($\chi^2 = 5.435$, $p = 0.020$) in the form of confluent hypointense abnormalities (Figure 1). Notably, this was also observed in one of the two iAD cases that received placebo.

Areas with white matter rarefaction on ex vivo GRE MRI, including one of the two iAD cases who received placebo, corresponded to granular neuropil changes on histopathology. Similar observations have previously been reported in banked tissue samples that were fixed in formalin for more than six years.[39] In our hands, these neuropil changes were positive for extracellular calcium. Therefore, as a control experiment, we stained paraffin sections taken from occipital samples in the same iAD cases that were embedded shortly after autopsy within 4 weeks of formalin fixation. Von Kossa staining revealed absence of extracellular calcium deposits. Notably, we did observe intracellular calcium deposits in one case (iAD case #7) in the shorter fixed tissue. Based on these observations, we concluded that longer fixation in formalin likely contributed to extracellular calcium depositions, which appeared on ex vivo MRI as white matter rarefaction.

Findings in non-AD control cases

To further control for prolonged formalin fixation, we scanned an additional six non-AD cases that had been fixed for >20 years under the same conditions as the iAD cases (Supplemental Table 1). Interestingly, we observed in 1/6 cases similar curvilinear hypointense abnormalities resembling cortical superficial siderosis (Supplemental Figure 3). On histopathology, however, we did not find the same extensive intracellular mineralization as in the iAD cases. Instead, only few extracellular iron depositions could be found. Notably, in all six cases we observed extracellular calcium deposits in both cortex and white matter.

Based on these observations, we excluded all extracellular iron and calcium deposits from further analysis, and solely focused on the intracellular alterations, which were only observed in the iAD cases in cortical areas with hemorrhagic lesions on ex vivo MRI.

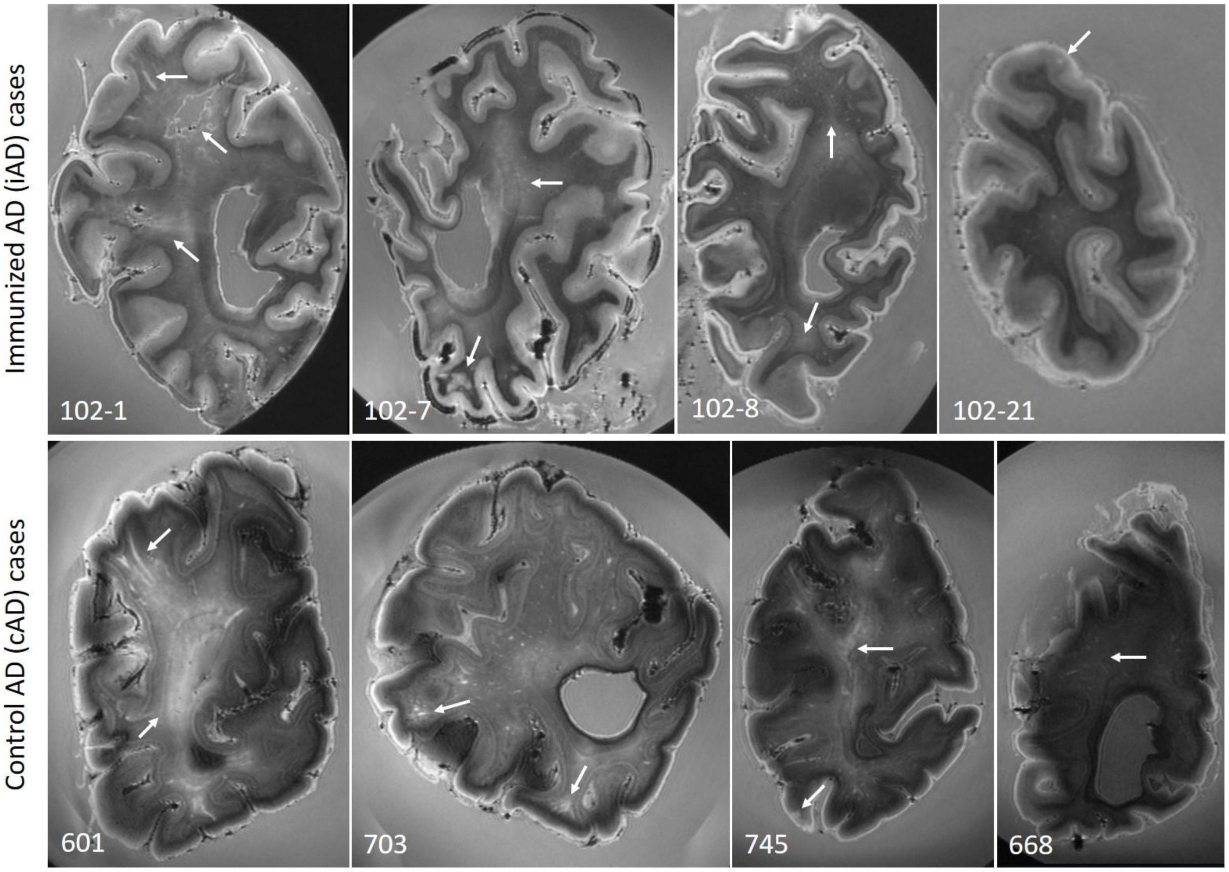

Supplemental Figure 1. Ex vivo turbo spin echo 7 T MRI is comparable between iAD and cAD cases.

Top row shows examples of hyperintensities (arrows) that were observed on ex vivo turbo spin echo (TSE) 7 T MRI scans of formalin-fixed coronal slices of iAD cases, representing enlarged perivascular spaces, white matter hyperintensities, and microinfarcts. Similar abnormalities were observed in cAD cases (arrows, bottom row).

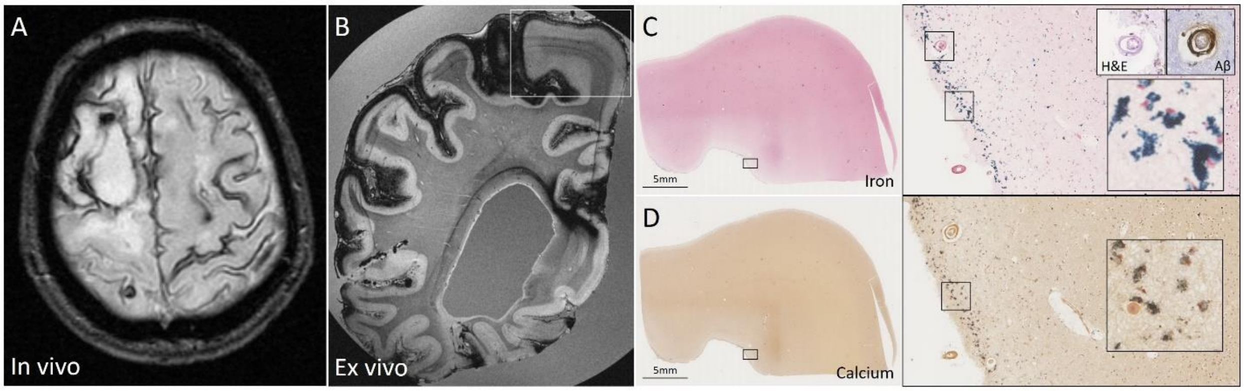

Supplemental Figure 2. Severe cortical superficial siderosis in a case with pathologically confirmed cerebral amyloid angiopathy.

Disseminated cortical superficial siderosis can be seen on an in vivo gradient echo (GRE) clinical MRI scan in a patient with a diagnosis of probable cerebral amyloid angiopathy (CAA) (A). On the ex vivo GRE 7 T MRI scan of a formalin fixed brain slice of the same case, cortical superficial siderosis was found in the same areas of the brain, resembling the curvilinear cortical hypointensities seen in the iAD cases (B). Note that in this case the hypointensities extend further into the sulci. On neuropathological examination, hemosiderin was found in the superficial layers of the cortex. Corresponding histopathological sections confirmed the presence of intracellular iron depositions (C) and intracellular calcium (D) in the cortex. This case had severe leptomeningeal CAA and concentric vessel wall splitting (see inset), as previously reported.[11]

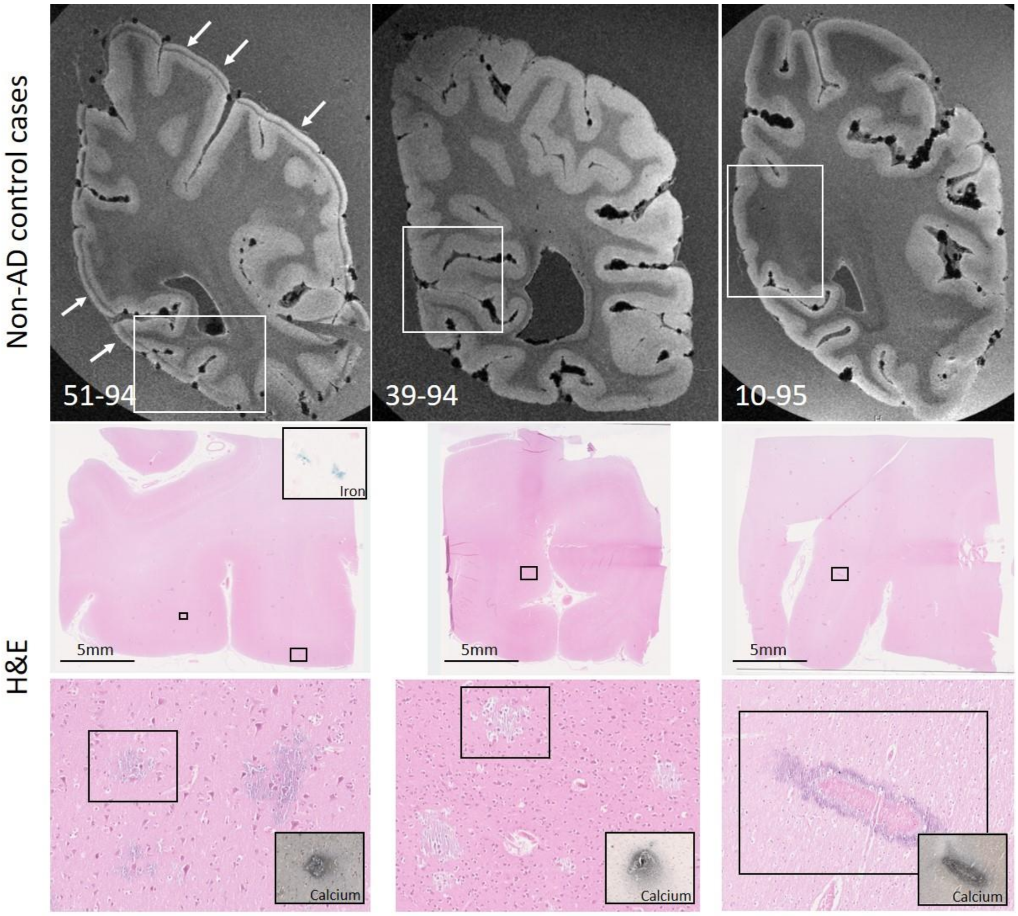

Supplemental Figure 3. Findings in non-AD control cases.

Top row shows curvilinear cortical hypointensities (arrows) that were observed on the ex vivo gradient echo (GRE) 7 T MRI scan of a formalin-fixed coronal slice of one of six non-AD control cases No abnormalities were observed in the other non-AD control cases. In the case with curvilinear cortical hypointensities on ex vivo MRI the Perls' Prussian blue-stained section was positive for iron, but the pattern differed, and the staining was much more subtle compared to the iAD cases. No intracellular iron or calcium depositions were found in these areas. Additional histopathological examination revealed widespread calcium-positive granular neuropil changes, indicative of prolonged formalin fixation in all cases. Note that these tissue alterations were not related to signal abnormalities on ex vivo MRI.

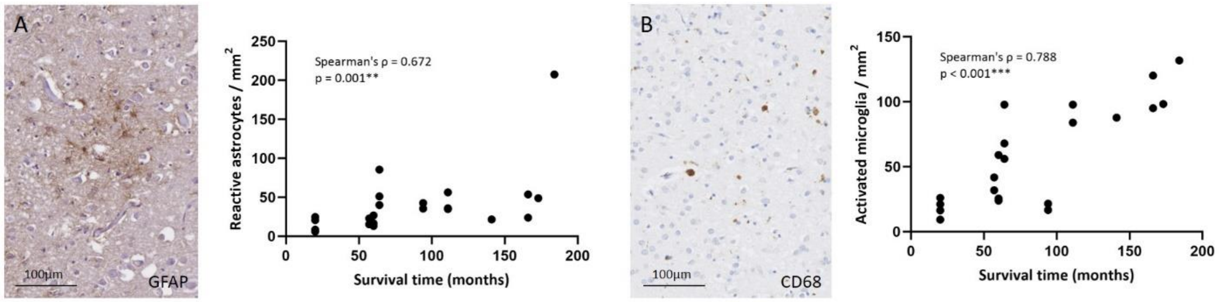

Supplemental Figure 4. Longer survival time is associated with greater number of reactive astrocytes and phagocytic microglia in iAD cases.

A representative example of reactive astrocytes on a section that underwent immunohistochemistry against glial fibrillary acidic protein (GFAP) is shown in (A). Within the iAD cases (*n* = 22 samples in 10 cases) post-immunization survival time was positively correlated with density of reactive astrocytes in the cortex (Spearman's ρ = 0.672, p = 0.001). A representative example of activated/phagocytic microglia on a section that underwent immunohistochemistry against CD68 is shown in (B). Within the iAD cases post-immunization survival time was positively correlated with density of activated/phagocytic microglia in the cortex (Spearman's ρ = 0.788, p < 0.001). ** p < 0.01, *** p < 0.001.

Supplemental Table 1. Characteristics on the non-AD control cases and ex vivo 7 T MRI findings.

Case ID	Sex (M/F)	Age at death (years)	Time of death (year)	Cause of death	Neuropathology findings	Post-mortem interval (hours)	Ex vivo 7 T MRI findings
39-94	M	71	1994	Chronic ischemic heart disease	Leptomeningeal fibrosis, neocortical atrophy and ventricular dilatation, control brain	47	Unremarkable
51-94	F	64	1994	Acute myocardial infarction	Normal brain	44	Disseminated curvilinear cortical hypointensities on GRE
10-95	F	61	1995	Acute myocardial infarction	Normal brain	48	Unremarkable
61-95	F	87	1995	Heart failure	Moderately severe large vessel disease	48	Unremarkable
69-95	F	81	1995	Heart failure	Normal brain	30	Unremarkable
4-97	M	77	1997	Acute myocardial infarction	Normal brain	0	Unremarkable
